# Impact of the Abdominal Drawing-In Maneuver on Spinal Extensor Muscle Activity: A Randomized Controlled Double-Blind Trial Involving Individuals with Non-Specific Low Back Pain

**DOI:** 10.3390/ijerph21121675

**Published:** 2024-12-16

**Authors:** Caglar Soylu, Emre Serdar Atalay, Duygu Turker, Tezel Yildirim Sahan, Necmiye Un Yildirim

**Affiliations:** Gulhane Faculty of Physical Therapy and Rehabilitation, University of Health Sciences, 06010 Ankara, Turkey; emreserdar.atalay@sbu.edu.tr (E.S.A.); duygu.turker@sbu.edu.tr (D.T.); tezelyildirim.sahan@sbu.edu.tr (T.Y.S.); necmiyeun.yildirim@sbu.edu.tr (N.U.Y.)

**Keywords:** abdominal drawing-in maneuver, electromyography, non-specific low back pain, spinal muscle activation, rehabilitation exercises

## Abstract

Non-specific low back pain (NSLBP) is a common musculoskeletal issue that can limit function and reduce the patient’s quality of life. Enhancing spinal stabilizer muscle activity through targeted exercises may help improve spinal alignment and alleviate NSLBP symptoms. This study aimed to investigate whether incorporating the abdominal drawing-in maneuver (ADIM) into selected low back exercises influences the electromyographic (EMG) activity of key spinal extensor muscles. Forty participants with NSLBP (n = 29 female and n = 11 male; mean age = 21.42 ± 1.07 years; BMI = 20.65 ± 2.08 kg/m^2^; 80% right-side dominant) performed three exercises, prone trunk extension, superman, and unstable superman, with and without the ADIM. The EMG amplitudes of the iliocostalis lumborum pars lumborum (ICL), iliocostalis lumborum pars thoracis (ICT), and longissimus thoracis (LT) were measured. A cross-sectional observational study design was employed. Significant main effects were observed for both exercise types and the ADIM on the EMG amplitudes of the ICL, ICT, and LT (ICL: F1,14 = 82.69–114.23, *p* < 0.001, η^2^ ≥ 0.88; ICT: F1,14 = 100.69–117.13, *p* < 0.001, η^2^ ≥ 0.90; LT: F1,14 = 62.69–74.88, *p* < 0.001, η^2^ ≥ 0.81). Under ADIM conditions, the ICL activity decreased significantly (right ICL mean difference: 14.06–20.02; left ICL: 13.06–21.32; *p* < 0.001), while the ICT and LT activity increased (ICT mean difference: 6.45–8.89; LT: 9.37–12.13; *p* < 0.001). These changes were most pronounced during the unstable superman exercise (*p* < 0.01). Integrating the ADIM into specific low back exercises can differentially modulate spinal extensor muscle activity. In particular, the unstable superman exercise demonstrated the greatest changes in the EMG amplitudes. These findings support the inclusion of the ADIM in exercise programs aimed at improving spinal stability and may have implications for NSLBP management. Future research should examine the effects of the ADIM in populations with varying experience levels to enhance its generalizability and refine the clinical recommendations.

## 1. Introduction

The interplay between spinal alignment and the multifaceted nature of low back pain (LBP) underscores the fundamental importance of maintaining optimal spinal posture. Based on seminal studies, the narrative emphasizes how the curvature of the thoracic and lumbar regions is shaped by the biomechanical properties of the spinal ligaments, as well as the synergistic and antagonistic forces of the surrounding musculature [1]. Deviations from ideal spinal curvatures, manifested as increased lordosis or marked kyphotic postures, are not only a precursor to chronic LBP, but are also understood to disrupt normal lumbar motion patterns [2]. These changes can trigger conditions such as thoracic flexion syndrome and can complicate shoulder mechanics [3]. This can lead to problems such as shoulder flexion dysfunction [4]. In response to these challenges, the strategic strengthening of the thoracic and lumbar spine’s extensor musculature emerges as a pivotal intervention. This approved approach aims to correct excessive thoracic kyphosis and lumbar lordosis, reduce spinal disorders, and prevent subsequent complications [5]. The essence of this therapeutic strategy rests on identifying and implementing exercises that effectively bolster these critical spinal regions.

Despite the dominance of subjective assessments in the existing literature, evaluating how exercises reduce pain, enhance lumbar muscle strength, and alleviate disability, there remains a notable scarcity of studies employing objective metrics. Nonetheless, research examining specific interventions, such as Tai Chi and Pilates, has shown significant improvements in pain, function, and kinesiophobia, among individuals with chronic low back pain [6,7,8,9,10]. However, objective studies demonstrating decreased lumbar muscle activation on both symptomatic and asymptomatic sides in patients with chronic LBP are rare [11,12,13,14]. The criticality of targeted muscle activation is brought to the fore by Park et al. [12] and Kim et al. [13], who demonstrated that exercises like the prone trunk extension and the abdominal drawing-in maneuver during prone hip extension significantly modulate the activity of the thoracic and lumbar erector spinae muscles, as well as the gluteus maximus. Similarly, a study on the superman exercise revealed no significant difference in the activity levels of the longissimus and iliocostalis muscles between stable and unstable modalities [14]. Building on this foundation, the present study aims to delve deeper into the effects of the abdominal drawing-in maneuver (ADIM) on the activation of thoracic and lumbar erector spinae muscles throughout a variety of low back exercises in individuals with non-specific low back pain. This inquiry is twofold: firstly, to systematically assess the efficacy of different low back exercises in conjunction with the abdominal drawing-in maneuver for optimizing the engagement of specific muscle groups and, secondly, to distill actionable insights that could refine therapeutic approaches to ameliorating LBP. The hypothesis underpinning this study posits that integrating the abdominal drawing-in maneuver into selected low back exercises will significantly increase the activation of the thoracic and lumbar erector spinae muscles in individuals with non-specific low back pain.

## 2. Materials and Methods

### 2.1. Participations

The sample size for this investigation was determined using power analysis. Based on the results of a pilot study involving 10 subjects, a sample size of 40 subjects was required to achieve an effect size of 0.40 (calculated using a partial η^2^ of 0.20), with an alpha level of 0.05 and a power of 0.80. A total of 40 subjects with non-specific low back pain were recruited to meet the calculated sample size. Data collection for this study took place between May 2022 and April 2023 in a controlled laboratory setting at the Gulhane Faculty of Physiotherapy and Rehabilitation, University of Health Sciences. Participants were recruited through advertisements circulated at local clinics, community centers, and the university campus. The inclusion criteria were as follows: (1) current lumbar pain; (2) not participating in any regular flexibility or strengthening exercise programs; (3) no history of lumbar spine surgery; (4) a kyphosis angle of 20–45 degrees; and (5) a lumbar lordosis angle of 20–40 degrees. Participants were excluded if they had acute low back pain, scoliosis, recent spinal surgery, or a previous whiplash injury. This study was approved by the University of Health Sciences Scientific Research Ethics Board (approval number: 2022-141; date of approval: 21.04.2022) and was conducted in compliance with the Declaration of Helsinki. It was registered on the ClinicalTrials.gov Protocol Registration and Results System website (Clinical Trial No. NCT05748509). All participants provided written informed consent before the commencement of data collection.

In this study, randomization was conducted using a computer-generated randomization method to assign the participants to exercise conditions (with or without ADIM) in a random order. This approach aimed to minimize bias between the groups and enhance the reliability of the results. The study was designed as a double-blind trial, ensuring that both participants and data collectors were blinded to the group assignments. The participants were unaware of which exercise protocol they were performing, ensuring unbiased performance, while the data collectors did not have access to group allocation information, preventing potential bias during measurement and analysis.

### 2.2. EMG Recording and Data Analysis

The EMG data were collected using a Noraxon Ultium EMG sensor system (Noraxon USA, Inc., Scottsdale, AZ, USA; sampling frequency of 4000 Hz per channel; gain 1000 [signal to noise ratio; 1 μV root mean square (RMS)]; common mode rejection rate (CMRR) −100 dB; input impedance >100 mΩ). Any hair on the skin was removed and then the area was cleansed with an alcohol swab, before electrodes were connected to detect EMG signals [14]. The electrodes were positioned bilaterally on the iliocostalis lumborum pars lumborum (right ICL and left ICL) at the L3 level, midway between the lateral-most palpable border of the erector spinae and a vertical line through the posterosuperior iliac spine; on the longissimus thoracis (right LT and left LT) at the T9 level, midway between a line through the spinous process and a vertical line through the posterosuperior iliac spine, located approximately 5 cm laterally; and on the iliocostalis lumborum pars thoracis (right ICT and left ICT) at the T10 level, midway between the lateral-most palpable border of the erector spinae and a vertical line through the posterosuperior iliac spine [8]. The sample rate was set to 2000 Hz. Two filters were applied, including a band-pass filtering at rates from 10 to 500 Hz, using a first-order high-pass and fourth-order low-pass Butterworth filter to remove unacceptable artifacts and a notch filter (60 Hz) to eliminate noise. A moving 100 ms window was used to calculate the RMS values. The Noraxon MyoResearch XP program was used to process the data (version 3.16; Noraxon Inc, Scottsdale, AZ, USA). The data for each trial were expressed as a percentage of the calculated mean RMS of the maximal voluntary isometric contraction (MVIC) (%MVIC) and the mean %MVIC of 3 trials was used for the analysis [15].

### 2.3. Procedures

Each muscle underwent an MVIC and the EMG signal amplitude was recorded, in order to normalize the EMG data. The test postures were similar to those presented in books on manual muscle testing that physical therapists frequently employ, except in the case of the back muscles, where more manual resistance was used [16]. The maximum resistance was standardized by using the Lafayette Manual Muscle Tester device (Lafayette Instrument Company, Lafayette, IN, USA), which progressively increased the manual pressure applied and held for 5 s to ensure consistency and accuracy across the participants. With a 30 s break between each contraction, each muscle test was performed three times. Also, by examining the EMG amplitudes during the manual muscle tests, proper electrode placement was validated. When performing the MVIC for the erector spina muscles, the lower extremities were stabilized, while the prone trunk was extended to its full range of motion and resistance was applied in the upper thoracic area [8,13].

All the participants underwent a standardized 30 min training session to learn and practice the abdominal drawing-in maneuver (ADIM) prior to data collection. The training was conducted by experienced professionals who provided clear, standardized instructions on performing the maneuver. During the session, participants received real-time feedback to refine their technique and ensure consistent application. The session included visual demonstrations, verbal cues, and hands-on corrections, as needed. To assess mastery, participants were required to demonstrate the ADIM technique correctly multiple times under supervision. The aim of this standardized training process was to minimize variability in technique execution during the experimental procedures and ensure uniform application across all the participants. Each participant used a pressure biofeedback device to practice abdominal hollowing, while being informed about the function and pressure monitoring system of the device (Chattanooga Group, Hixson, TN, USA). The EMG activity was measured during lumbar extension exercises, performed with and without doing an ADIM. Each repeat of the exercise was kept in the isometric position for 10 s. A metronome was used to control the time and speed of movement. The exercises were performed at a controlled speed, synchronized with a metronome set to 60 beats per minute, with each phase lasting for three ticks (3 s). Specifically, the concentric phase, isometric phase, and eccentric phase were each performed over three ticks, ensuring consistent execution and reproducibility across all the participants. The exercises were carried out in a random order. To minimize the potential effects of fatigue on the EMG measurements, specific rest intervals were incorporated into the study protocol. A 30 s rest period was allowed between each repetition of the same exercise and a one-minute break was provided between different exercises within the same condition. Additionally, a three-minute rest interval was implemented between exercise conditions (with and without ADIM) to ensure the participants had sufficient time to recover. To further control for order effects, each participant performed the lumbar extension exercises in a randomized sequence, alternating between conditions. These measures were carefully designed to enhance the reliability and consistency of the EMG data, by reducing the influence of fatigue and ensuring equal recovery opportunities across all exercises and conditions [9,12].

### 2.4. Exercise Procedures

#### 2.4.1. Prone Trunk Extension Exercise

The participants performed the prone trunk extension exercise while lying face down on a plinth, extending their trunks to touch a horizontal bar positioned 10 cm above the plinth. An electronic metronome provided auditory cues to maintain a consistent tempo, ensuring controlled execution during the ascent (lumbar extension) and descent (return to neutral) phases. Two standardized forms of fixation were applied to enhance stability: adjustable straps were used to secure the popliteal fossa of the knees and the posterior superior iliac spine of the pelvic region, while an additional strap was placed at the thoracolumbar junction. These fixations were applied uniformly to all participants to ensure consistency and minimize variability during the exercise [14].

#### 2.4.2. Superman Exercise

The participants began this hyperextension exercise in a prone position, with neutral spine curvatures. Throughout the entire exercise session, hands and arms were held at the level of the temporal bone, feet flat on the floor, legs extended, and arms flexed. The participants were instructed to perform lumbar hyperextension (approximately 20 degrees) during the ascent phase and return to neutral (0 degrees) during the descent phase. The degree of lumbar hyperextension was monitored using the digital inclinometer feature of the EMG device, ensuring precise measurement and consistent execution across the participants [10].

#### 2.4.3. Unstable Superman Exercise

The same steps as the superman exercise were followed here, but with the range of motion performed under unstable conditions. A Swiss ball was placed under the participant’s umbilicus, ensuring instability during the exercise. The participants positioned their feet a shoulder-width apart, flat against a wall for support, and kept their legs fully extended. Throughout the exercise, hands were held at the level of the temporal bone, with arms extended. The degree of lumbar hyperextension (approximately 20 degrees during the ascent phase) was monitored using the digital inclinometer feature of the EMG device, ensuring precise measurements and consistent execution during the unstable condition [10]. To account for anthropometric differences between the participants, such as torso length and flexibility, the size and positioning of the Swiss ball were individually adjusted. The ball was positioned under the umbilicus to provide proper support, while maintaining instability, and adjustments were made to ensure each participant could perform the exercise comfortably and effectively. Flexibility was also considered, ensuring the position allowed the participants to complete the movement without strain, while maintaining the proper form. The accuracy of the positioning and movement was monitored through visual assessment and the digital inclinometer feature of the EMG device, ensuring consistent lumbar hyperextension angles and minimizing variability across the participants.

All the lumbar extension workouts included the ADIM, which was carried out under the supervision of a pressure biofeedback device (PBFU; Chattanooga Group, Hixson, TN, USA). In the prone position, the PBFU was placed beneath the lower abdomen of the participants, and its position was adjusted individually to accommodate different body types, ensuring accurate and reliable readings. As soon as the metronome began, individuals initiated the ADIM, with the pressure set to 70 mmHg. They reduced the pressure to 60 mmHg and maintained it at this level throughout the exercise. The data collected within pressure changes of ±5 mmHg were used for the statistical analysis, with this threshold selected based on its alignment with prior research demonstrating its sensitivity in regard to detecting subtle variations in abdominal engagement during stabilization exercises. This adjustment ensured consistency and minimized variability in the dataset [12].

### 2.5. Statistical Analysis

All the analyses were conducted using the SPSS software (ver. 26.0; IBM, Armonk, NY, USA). The Kolmogorov–Smirnov test was used to confirm that the data were distributed normally. Four separate 2 × 2 analyses of variance determined the main and interaction effects for each tested muscle. The within-subject factor was the condition (two levels: with and without an ADIM). The statistical significance level was set at 0.05.

## 3. Results

**Figure 1:**
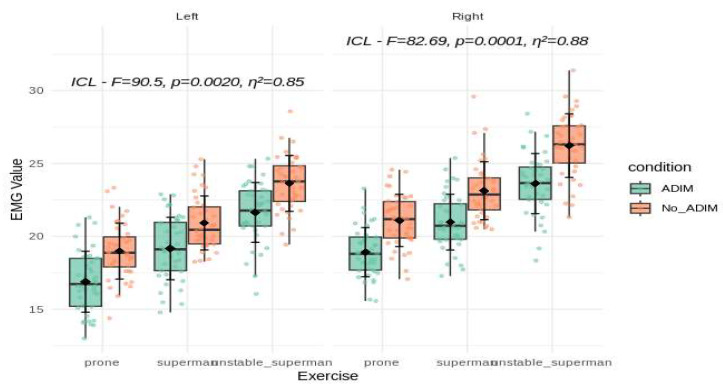
A comparison of the main effect on ICL muscle activity during the three different lower back exercises, with and without ADIM (mean ± SD). The mean of the measurements obtained for each exercise and the mean of their differences are presented with the *p*-value. ICL: iliocostalis lumborum pars lumborum; EMG value represents %MVIC: the percentage of maximal voluntary isometric contraction; ADIM: abdominal drawing-in maneuver.

**Figure 2:**
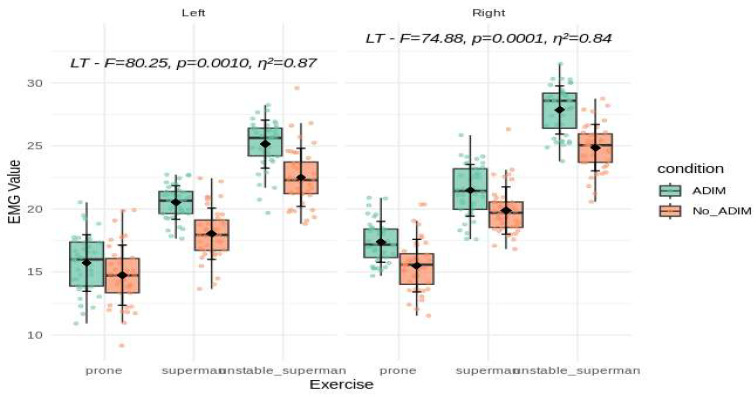
A comparison of the main effect on ICT muscle activity during the three different lower back exercises, with and without ADIM (mean ± SD). The mean of the measurements obtained for each exercise and the mean of their differences are presented with the *p*-value. ICT: iliocostalis lumborum pars thoracis; EMG value represents %MVIC: the percentage of maximal voluntary isometric contraction; ADIM: abdominal drawing-in maneuver.

**Figure 3:**
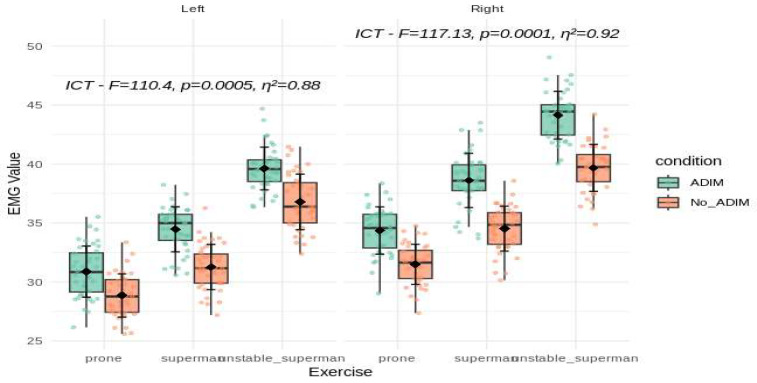
A comparison of the main effect on LT muscle activity during the three different lower back exercises, with and without ADIM (mean ± SD). The mean of the measurements obtained for each exercise and the mean of their differences are presented with the *p*-value. LT: longissimus thoracis; EMG value represents %MVIC: the percentage of maximal voluntary isometric contraction; ADIM: abdominal drawing-in maneuver.

A total of 40 participants with non-specific low back pain were included in this study, with 73.3% (n = 29) being female and 26.7% (n = 11) being male. All data are presented as the mean ± standard deviation, and statistically significant differences are indicated in Figure 1, Figure 2 and Figure 3. Both the right and left sides were analyzed separately, allowing for direct comparisons of muscle activation under varying exercises and conditions.

For the iliocostalis lumborum pars lumborum (ICL), significant main effects of the exercise type and the addition of the ADIM were observed on both sides, although with slightly different effect magnitudes. On the left side, the ICL showed a strong effect (F = 90.5, *p* = 0.0020, η^2^ = 0.85), while on the right side the effect remained robust (F = 82.69, *p* = 0.0001, η^2^ = 0.88). The descriptive data supported these findings: on the left side, the ICL EMG values increased from prone (17.9 ± 2.26) to superman (20.0 ± 2.18) and further during unstable superman (22.6 ± 2.21). On the right side, the ICL EMG values followed a similar upward trend from prone (20.0 ± 2.05) to superman (22.1 ± 2.22) and unstable superman (24.9 ± 2.48). The ADIM consistently enhanced the ICL activity across these exercises (Figure 1).

The iliocostalis lumborum pars thoracis (ICT) muscle displayed even more pronounced differences. On the left side, the ICT showed significant changes (F = 110.4, *p* = 0.0005, η^2^ = 0.88), while on the right side the effect size was slightly higher (F = 117.13, *p* = 0.0001, η^2^ = 0.92). The mean ± SD values illustrate these patterns: on the left side, the ICT EMG amplitude rose from prone (29.9 ± 2.24) through superman (32.9 ± 2.49) to unstable superman (38.2 ± 2.53), and on the right side from prone (32.9 ± 2.34) to superman (36.6 ± 2.93) and unstable superman (41.9 ± 3.00). The unstable superman exercise produced the highest ICT values on both sides, especially under ADIM conditions (Figure 2).

For the longissimus thoracis (LT) muscle, significant main effects were also apparent. On the left side, the LT activity showed substantial changes (F = 80.25, *p* = 0.0010, η^2^ = 0.87), while the right side also revealed strong effects (F = 74.88, *p* = 0.0001, η^2^ = 0.84). The left side LT EMG values increased from prone (15.2 ± 2.35) to superman (19.3 ± 2.12) and reached their peak during unstable superman (23.8 ± 2.48). On the right side, similarly, the LT activity escalated from prone (16.4 ± 2.09) to superman (20.7 ± 2.11) and attained its highest level during the unstable superman exercise (26.4 ± 2.40). As with the ICL and ICT, the more challenging exercises elicited greater LT activation, and the ADIM further enhanced these responses (Figure 3).

## 4. Discussion

This study provides extensive analysis of the effects of the ADIM on EMG activity in key spinal stabilizing muscles, including the iliocostalis lumborum pars thoracis, iliocostalis lumborum pars lumborum, and longissimus thoracis, during various exercises performed by individuals with NSLBP. The results demonstrated significant modulation of muscle activation patterns, with the ADIM enhancing the activation levels across these muscles. Among the exercises studied, the unstable superman exercise produced the highest activation levels, particularly when performed with the ADIM. This finding aligns with the concept that instability during exercises amplifies neuromuscular engagement and core stability. Research by Behm et al. [17] supports these observations, showing that exercises performed on unstable surfaces increase the neuromuscular demands and proprioceptive feedback, thus improving functional stability and core strength.

This study highlighted that the ADIM enhances thoracic muscle activation, while reducing lumbar strain, demonstrating its role in selectively recruiting deep stabilizing muscles. These findings align with Kim et al. [18], who showed that the ADIM increased thoracic extensor muscle activity during the prone trunk extension, effectively minimizing compensatory lumbar muscle engagement. Further, Kim et al. [19] compared the prone trunk extension with the four-point kneeling arm and leg lift exercises and found that the prone trunk extension elicited higher activation of the superficial lumbar multifidus. These results emphasize the ADIM’s clinical utility in addressing region-specific deficits. For instance, patients with thoracic kyphosis may benefit from exercises focusing on thoracic extensors, while those with lumbar hyperlordosis can achieve better outcomes with lumbar stabilization exercises that reduce unnecessary thoracic involvement.

Another critical finding was the variability in iliocostalis lumborum pars lumborum activation depending on the exercise type. The prone trunk extension elicited moderate activation, while the superman and unstable superman exercises showed progressively higher engagement. This variability reflects the functional demands of these exercises, with dynamic movements requiring greater stabilization efforts. Ishida et al. [20] reported similar results, highlighting the localized recruitment of lumbar stabilizers during dynamic tasks with the ADIM. Hwang and Park [21] demonstrated that graded superman exercises with the ADIM significantly increased lumbar multifidus thickness, while reducing perceived exertion. This suggests that the ADIM improves the efficiency of spinal stabilizers, enabling sustained activation with less effort, which is crucial for patients with NSLBP, who often experience muscle fatigue during prolonged tasks. Tailoring exercise selection based on individual needs is essential to maximize rehabilitation outcomes, especially for patients with NSLBP.

This study also demonstrated the ADIM’s significant role in reducing muscle fatigue. Barbosa et al. [22] and Oh et al. [23] observed similar outcomes, showing that the ADIM enhances endurance without inducing hypertrophy, making it particularly relevant for patients with chronic low back pain, who require prolonged activation of spinal stabilizers during daily activities. The reduction in fatigue ensures sustained muscle engagement during functional tasks, such as prolonged sitting or standing, thereby improving functional performance and reducing discomfort. This is particularly important for individuals with NSLBP, as the deconditioning of stabilizing muscles is a common contributing factor to their pain.

The incorporation of the ADIM into exercises performed on unstable platforms, such as the unstable superman, was shown to provide additional benefits. These exercises increased the proprioceptive feedback and dynamic control, enhancing neuromuscular activation and core control. Carvalheiro et al. [14] highlighted the benefits of instability-based exercises in improving core strength and balance, reinforcing the findings in this study. The unstable superman exercise, as the most demanding in this study, is particularly valuable for patients in the advanced stages of rehabilitation. However, its intensity necessitates careful progression, beginning with stable exercises to build foundational strength and control.

The ADIM also demonstrated differential effects on muscle activation during prone hip extension exercises. Lee et al. [24] found that the ADIM improved the activation of the posterior oblique sling, which includes the gluteus maximus and contralateral latissimus dorsi. This coordinated activation supports pelvic and spinal stability, further highlighting the ADIM’s utility in addressing movement inefficiencies and compensation often seen in patients with NSLBP.

The differential effects of the ADIM on thoracic and lumbar stabilization underscore its utility in targeted rehabilitation. Park et al. [25] demonstrated that the ADIM reduces lumbar strain by limiting anterior pelvic tilt and pelvic rotation, while enhancing hamstring activation during prone knee flexion. This allows clinicians to tailor exercises to patient-specific needs. The prone trunk extension with the ADIM can improve thoracic stability for patients with kyphosis, while the prone hip extension with the ADIM is more effective for lumbar stabilization. The ADIM’s ability to balance muscle activation and reduce compensatory movements highlights its value in improving movement efficiency and addressing biomechanical deficits in low back pain rehabilitation.

The ADIM’s ability to reduce compensatory patterns during functional activities further highlights its clinical relevance. Oh et al. [23] demonstrated that the ADIM minimizes anterior pelvic tilt during dynamic movements, promoting proper alignment and muscle recruitment. Similarly, Cruz-Diaz et al. [26] reported that stabilization exercises incorporating the ADIM improved functional outcomes in NSLBP patients, reducing pain and enhancing their quality of life. These findings suggest that the ADIM not only supports rehabilitation, but also prepares patients for real-world activities, such as lifting, walking, and transitioning between postures.

Despite its strengths, this study has certain limitations that should be addressed. The participant pool predominantly consisted of younger individuals, with limited representation of older age groups. Additionally, the study included more female participants and the reasons for the limited inclusion of male participants were not elaborated. These demographic constraints raise questions about the generalizability of the findings across genders and different age groups. Furthermore, while the study focused on immediate EMG outcomes, it did not assess the long-term effects, functional improvements, or pain reduction, which are crucial for understanding the broader impact of the ADIM. The absence of kinematic analysis also limits insights into movement mechanics and compensatory patterns. Future research should aim to include a more balanced representation of genders and age groups and evaluate whether these results can be generalized to diverse populations. Longitudinal studies incorporating biomechanical assessments are needed to explore the sustained benefits and functional improvements in both genders and across all age ranges.

## 5. Conclusions

In conclusion, this study highlights the significant benefits of the ADIM in enhancing spinal stabilizer activation, improving neuromuscular efficiency, and reducing muscle fatigue in individuals with NSLBP. The ADIM effectively recruits deep thoracic and lumbar stabilizing muscles, such as the iliocostalis and longissimus thoracis, without overloading the lumbar spine. Notable variations in muscle activation were observed across the different exercises, with the unstable superman exercise eliciting the highest activation levels. This finding underscores its potential in advanced rehabilitation protocols to improve core strength, dynamic control, and functional performance. The ADIM’s ability to reduce fatigue and compensatory movements further supports its utility in sustaining functional stability during daily activities, such as prolonged sitting or standing.

Clinically, the ADIM should be integrated as a cornerstone into NSLBP rehabilitation protocols. Beginning with foundational exercises like the prone trunk extension and superman, patients can gradually progress to instability-based exercises, such as the unstable superman, which enhance core strength and dynamic stability. This progression ensures safety, while maximizing the therapeutic benefits. Moreover, incorporating the ADIM into rehabilitation strategies can optimize muscle function, reduce fatigue, and improve patient outcomes. Future research should explore its long-term effects and applicability in diverse populations, incorporating kinematic analyses and longitudinal outcomes to better understand its role. With its demonstrated ability to improve spinal stability and functional performance, the ADIM offers a targeted and adaptable solution for effectively managing NSLBP and rehabilitation.

## Data Availability

The original contributions presented in this study are included in the article.
